# The Impact of Transcatheter Aortic Valve Implantation (TAVI) on Serum Apelin Levels in Patients with Aortic Valvular Stenosis

**DOI:** 10.21470/1678-9741-2020-0208

**Published:** 2021

**Authors:** Mehmet Kucukosmanoglu, Seyda Sahin, Orsan Deniz Urgun, Arafat Yildirim, Salih Kilic, Omer Sen, Ibrahim Halil Kurt

**Affiliations:** 1 Department of Cardiology, Health Sciences University, Adana Training and Research Hospital, Adana, Turkey.

**Keywords:** Apelin, Aortic Valve Stenosis, Transcatheter Aortic Valve Implantation, Echocardiography

## Abstract

**Introduction:**

In this study, we aimed to investigate the impact of transcatheter aortic valve implantation (TAVI) on serum apelin levels in patients with severe symptomatic aortic valve stenosis (AS).

**Methods:**

Forty-six consecutive patients (76.9±7.4 years, n=27 women) who underwent TAVI and 45 age- and sex-matched control subjects were included in the study. Echocardiographic parameters, serum apelin, pro-brain natriuretic peptide (Pro-BNP), and troponin I levels were compared between the groups. In addition, the preprocedural and first-month follow-up echocardiographic parameters and serum apelin values of TAVI patients were compared.

**Results:**

Serum median troponin I and Pro-BNP levels were significantly higher and serum apelin levels were significantly lower in TAVI patients before TAVI procedure than in the control subjects (*P*<0.001, for all). Median troponin I and Pro-BNP levels were significantly decreased and apelin levels were significantly increased after TAVI procedure compared to the peri-procedural levels. There was a significant and moderate negative correlation between Pro-BNP and apelin levels measured before and after TAVI procedure. A statistically significant and strong negative correlation was found between aortic valve area and Pro-BNP level before TAVI procedure, while a statistically significant but weak positive correlation was found between valve area and apelin level.

**Conclusion:**

In our study, apelin levels were significantly lower and Pro-BNP levels were higher in AS patients compared with the control group. Moreover, after TAVI procedure, a significant increase in apelin levels and a significant decrease in Pro-BNP levels were observed. There was also a negative and moderate correlation between apelin and Pro-BNP levels.

**Table:** 

Abbreviations, acronyms & symbols				
**ACE-i**	**= Angiotensin-converting enzyme inhibitor**		**HT**	**= Hypertension**
**ARB**	**= Angiotensin receptor blockers**		**IVRT**	**= Isovolumetric relaxation time**
**AS**	**= Aortic valve stenosis**		**LDL-C**	**= Low-density lipoprotein cholesterol**
**AVA**	**= Aortic valve area**		**LV**	**= Left ventricular**
**BMI**	**= Body mass index**		**LVEF**	**= Left ventricular ejection fraction**
**CI**	**= Confidence interval**		**Pro-BNP**	**= Pro-brain natriuretic peptide**
**HDL-C**	**= High-density lipoprotein cholesterol**		**TAVI**	**= Transcatheter aortic valve implantation**
**HFrLVEF**	**= Heart failure with reduced left ventricular ejection fraction**		**TTE**	**= Transthoracic echocardiography**

## INTRODUCTION

Aortic valve stenosis (AS) is the most common heart valve disease in Europe and North America, requiring the most surgical or catheter intervention, and its incidence increases with advancing age. Calcific AS, especially observed in patients older than 65 years of age, is the most common form of this disease^1^. Transcatheter aortic valve implantation (TAVI) is an alternative treatment for patients with severe AS who cannot undergo surgical aortic valve replacement or are at high risk^2-4^.

Apelin is an adipokine isolated from cattle gastric juice in 1998 by Tatemato et al.^5^. Preproapelin, a precursor of apelin, which was also isolated from adipose tissue in 2005 and accepted as a new member of the adipose tissue family, was detected in many tissues, while the biologically active apelin was detected in the epithelial cells of the gastric mucosa, myocardial and endocardial tissues, and the endothelium of large and small vessels^6,7^. Data from previous studies indicate that apelin may have significant regulatory effects on myocardial contraction, blood pressure, angioneogenesis, and fluid balance in addition to inhibiting apoptosis^8-10^. Studies have shown that serum apelin levels decrease in many serious cardiovascular diseases^9,11,12^. To our best knowledge, there are no studies in the literature investigating serum apelin levels in patients undergoing TAVI for AS. In the present study, we aimed to investigate the effect of TAVI on serum apelin levels in patients with symptomatic AS.

## METHODS

### Patient Population

Forty-six consecutive patients who underwent TAVI in our clinic, between July 2019 and December 2019, for severe symptomatic calcific AS and 45 age- and sex-matched, non-aortic stenosis patients were prospectively included in the study. Patients with history of severe coronary artery disease, peripheral artery disease, acute coronary syndrome, atrial fibrillation, congenital heart disease, myocarditis, pericarditis, gouty arthritis, use of diuretic drugs, use of xanthine oxidase inhibitor and hepatotoxic drugs, acute or chronic renal dysfunction, primary or secondary hyperparathyroidism, malignancies, acute or chronic inflammatory diseases, and active infections were excluded from study.

The study protocol was approved by the local ethics committee and informed consent was obtained from all patients included in the study.

Patients’ baseline medical and demographic characteristics were recorded before TAVI procedure. In addition, before the procedure, venous blood samples were collected from all patients for routine blood parameters. Serum apelin and serum pro-brain natriuretic peptide (Pro-BNP) levels of TAVI patients were measured before TAVI procedure and one month after it. Also, serum apelin and serum Pro-BNP levels of control subjects were measured and compared with the levels of before TAVI procedure.

The first-month control of the patients was carried out by the physician performing the TAVI procedure at the outpatient clinic.

### Definitions

Hypertension (HT) was defined as the patient's systolic and diastolic blood pressure being > 140/90 mmHg measured at rest and/or using any antihypertensive drug. Diabetes mellitus was defined as the patient's measured fasting blood glucose > 126 mg/dL, the measured blood glucose > 200 mg/dl at any time and/or taking antidiabetic medication. Dyslipidemia was defined as the total cholesterol level ≥ 200 mg/dL and/or using any lipid-lowering treatment. Smoking was defined as having one or more cigarettes in a day for at least one year.

### Echocardiography

All patients included in the study were evaluated by transthoracic echocardiography (TTE) one day before and one month after the TAVI procedure. Aortic valve functions were performed according to the recommendations of the Valve Academic Research Consortium-2, or VARC-2, before the procedure and at the follow-up^13^. Standard echocardiographic imaging (two-dimensional, M-mode, conventional Doppler and color Doppler) was performed by two experienced cardiologists who did not know about the patient's clinical presentation and laboratory parameters, using a dedicated ultrasound machine (ACUSON SC2000; Ultrasound, Siemens) with a 2.5-3.5 and Z6 MHz transducer.

### Blood Sample Collection and Analyses

To determine serum apelin levels, 10 ml venous blood samples were collected from patients before TAVI procedure and one month after it. Likewise, 10 ml venous blood samples were collected from the control group. The venous blood samples of the patients and controls were centrifuged at 4,000 rpm for 10 minutes. Sufficient plasma was placed in Eppendorf Tubes^®^ and incubated at -80 ºC until analyzed. Serum apelin levels were measured using a commercially available enzyme-linked immunosorbent assay, also known as ELISA, kit (Cusabio Biotech Co., Wuhan, People’s Republic of China).

### Statistical Analysis

Normal distribution of the continuous parameters *(i.e*., age, troponin, apelin) was given as mean (± standard deviation). Those without normal distribution were given as median (interquartiles 25^th^-75^th^). Categorical variables (*i.e*., sex, HT) were given as numbers and percentages (%). Continuous parameters with normal distribution were compared with Student's *t*-test, whereas non-normally distributed parameters were compared with Mann-Whitney U test. Categorical parameters were compared with Chi-square test or Fisher's exact test. Regarding the parameters measured before and after TAVI procedure (such as apelin and Pro-BNP), those with normal distribution were compared with the paired Student's *t*-test and those without normal distribution were compared with the Wilcoxon test. Spearman or Pearson correlation analyses were performed to show the relationship between the parameters. Data analysis was performed with IBM Corp. Released 2011, IBM SPSS Statistics for Windows, Version 20.0, Armonk, NY: IBM Corp. A *P*<0.05 was determined as statistically significant.

## RESULTS

Forty-six patients (n=27 women, 60%) with a mean age of 76.9±7.4 years who underwent TAVI and age- and sex-matched 45 controls subjects (mean age 77.9±9.5 years) were included in the study. The demographic and medical characteristics of both groups are summarized in Table 1. There was no statistically significant difference between the groups in terms of clinical characteristics, drug use rates, and routine laboratory parameters.

**Table 1:** Comparison between the demographic and medical characteristics and echocardiographic parameters of the groups.

Variables	TAVI (n=46)	Control (n=45)	P-value
Age, mean	76.7±7.4	77.9±9.5	0.320
Female, % (n)	60 (27)	66.7 (30)	0.512
BMI (kg/m^2^)	27.6±3.9	26.7±4.2	0.322
Diabetes mellitus, % (n)	26.7 (12)	28.9 (13)	0.814
Hypertension, % (n)	66.7 (30)	68.9 (31)	0.822
Hyperlipidemia, % (n)	35.56 (16)	40.0 (18)	0.664
Smoking, % (n)	26.7 (12)	28.9 (13)	0.814
Coronary artery disease, % (n)	51.1 (23)	22 (48.9)	0.833
Stroke, % (n)	4.4 (2)	6.7 (3)	0.645
Beta-blocker, % (n)	42.2 (19)	48.9 (22)	0.525
ACE-i/ARB, % (n)	57.8 (26)	62.2 (28)	0.667
Statin, % (n)	44.4 (20)	40.0 (18)	0.670
Aspirin, % (n)	53.3 (24)	51.1 (23)	0.833
Diuretics, % (n)	8.9 (4)	13.3 (6)	0.502
Fasting blood glucose, mg/dL	121±34	122±36	0.865
Urea, mg/dL	47.4±23	43.6±34	0.541
Creatinine, mg/dL	1.05±0.97	0.87±0.1	0.233
Uric acid, mg/dL	5.9±1.44	5.3±1.32	0.135
Total protein, g/dL	6.5±0.54	6.4±0.43	0.518
Albumin, g/dL	3.6±0.4	3.7±0.4	0.095
Total cholesterol, mg/dL	173±42	190±37	0.343
LDL-C, mg/dL	113±23	125±21	0.211
HDL-C, mg/dL	45±13	44±11	0.497
Triglyceride, mg/dL	129±64	125±43	0.775
Troponin I median (25^th^-75^th^), ng/ml	0.350 (0.240-0.470)	0.055 (0.016-0.203)	<0.001
Pro-BNP, median (25^th^-75^th^), ng/L	2455 (1160-3690)	32 (26-36)	<0.001
Apelin median (25^th^-75^th^), ng/ml	172 (150-207)	375 (340-417)	<0.001
LVEF, %	56.5±8.3	59.4±7.5	0.341
AVA, cm^2^	0.73±0.12	-	-
Mean gradient (mmHg)	50±10.7	-	-
E/A ratio	1.01±0.11	0.97±0.11	0.671
Septal/lateral e' (mean)	6.9±1.1	8.1±1.4	0.001
IVRT (msn)	104±6.6	81±5.3	<0.001
E/e' ratio	8.5±1.57	6.1±1.25	<0.001
LV mass index, g/m^2^	130±13.4	92±10.8	<0.001

ACE-i=angiotensin-converting enzyme inhibitor; ARB=angiotensin receptor blockers; AVA=aortic valve area; BMI=body mass index; HDL-C=high-density lipoprotein cholesterol; IVRT=isovolumetric relaxation time; LDL-C=Low-density lipoprotein cholesterol; LV=left ventricular; LVEF=left ventricular ejection fraction; Pro-BNP=pro-brain natriuretic peptide; TAVI=transcatheter aortic valve implantation

While median serum troponin I levels (0.350 ng/ml (0.240-0.470) *vs*. 0.055 ng/ml (0.016-0.203); *P*<0.001) and serum Pro-BNP levels (2455 (1160-3690) ng/L *vs*. 32 (26-36) ng/L; *P*<0.001) measured before TAVI procedure were significantly higher than those from the control group, serum apelin levels (172 (150-207) ng/ml *vs*. 375 (340-417) ng/ml; *P*<0.001) were significantly lower (Table 1).

**Fig. 1:**
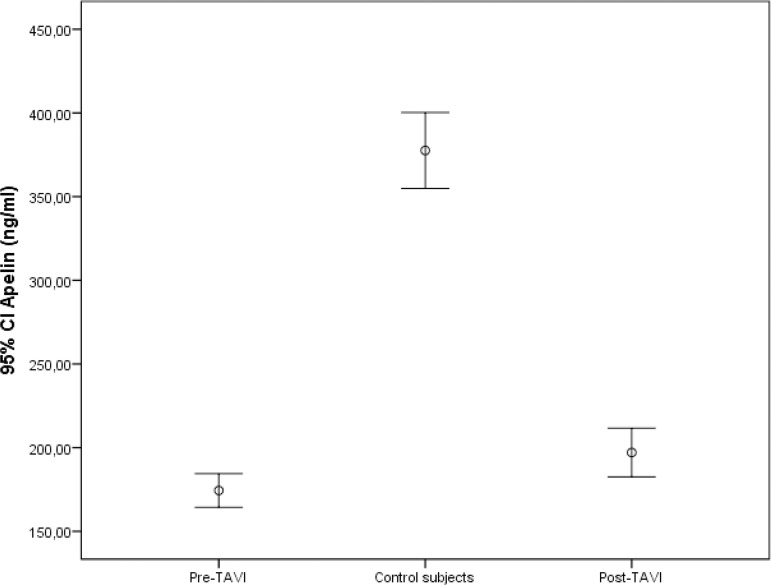
Comparison of serum apelin levels between control subjects and pre- and post-transcatheter aortic valve ımplantation (TAVI) procedure. CI=confidence interval

The mean isovolumetric relaxation time and E/e’ values of the patients detected by TTE before TAVI procedure were higher than those from the control group, whereas the mean septal and lateral e’ values were lower (Table 2). There was no significant change in left ventricular systolic function after TAVI procedure, but a significant improvement in diastolic function was observed (Table 3). The median serum Pro-BNP levels after TAVI were significantly reduced (2455 (1140-3770) ng/L *vs*. 1010 (460-1950) ng/L; *P*<0.001), while the median serum apelin levels were significantly increased (172 (150-207) ng/ml *vs*. 193 (180-220) ng/ml; *P*<0.001). In addition, the median serum apelin levels detected in the patients after TAVI procedure were significantly lower compared to the control group (193 (180-220) ng/ml *vs*. 375 (340-417) ng/ml; *P*<0.001) (Figure 1).

**Table 2:** Correlation between serum apelin and Pro-BNP levels and echocardiographic parameters.

Variables	r	*P*-value
**Apelin**		
Pro-BNP (pre-TAVI)	-0.460	0.001
Pro-BNP (post-TAVI)	-0.441	0.002
AVA	0.376	0.011
Mean gradient	-0.372	0.012
LV mass index	-0.844	< 0.001
Troponin I	-0.444	< 0.001
**Pro-BNP (pre-TAVI)**		
AVA	-0.803	< 0.001
Mean gradient	0.795	< 0.001
LV mass index	0.723	< 0.001
Troponin I	0.489	< 0.001

AVA=aortic valve area; LV=left ventricular; Pro-BNP=pro-brain natriuretic peptide; TAVI=transcatheter aortic valve implantation

**Table 3:** Comparison between the pre and post-TAVI echocardiographic parameters of the patients.

Variables	Pre-TAVI	Post-TAVI	P-value
E/A ratio	1.01±0.11	1.04±0.12	0.213
Septal-lateral e' (mean)	6.9±1.1	7.4±0.8	<0.001
IVRT (msn)	104±6.6	98.4±7.1	<0.001
E/e' ratio	8.5±1.57	7.1±1.02	<0.001
LV mass index, g/m^2^	130±13.4	129±12.8	0.194
LVEF, %	56.5±8.3	58±9.1	0.342

IVRT=isovolumetric relaxation time; LV=left ventricular; LVEF=left ventricular ejection fraction; TAVI=transcatheter aortic valve implantation

**Fig. 2:**
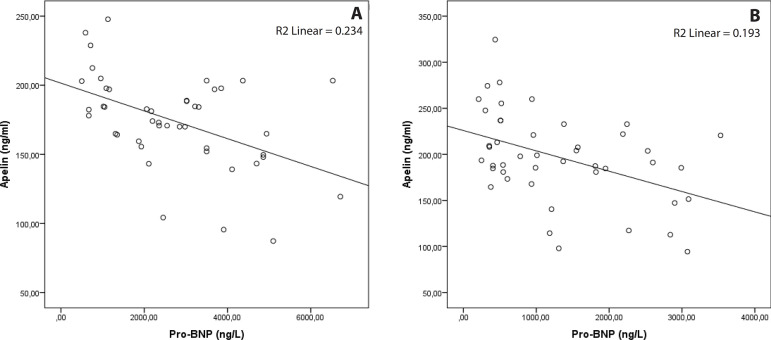
Correlation between serum pro-brain natriuretic peptide (Pro-BNP) and apelin levels pre- and post-transcatheter aortic valve implantation. A) Pre-procedure; B) post-procedure

There was a statistically significant, moderate, and negative relationship between serum Pro-BNP and serum apelin levels both before and after the TAVI procedure (Table 3) (Figure 2). There was a statistically significant, strong, and negative correlation between aortic valve area and serum Pro-BNP levels before the TAVI procedure, while a statistically significant, moderate, and positive correlation was found between valve area and serum apelin levels. There was also a statistically significant, strong, and positive correlation between aortic valve mean gradient and serum Pro-BNP levels, while a statistically significant, moderate, and negative correlation was found between aortic valve mean gradient and serum apelin levels (Table 3). There was a negative, strong correlation between serum apelin levels and left ventricular mass index before TAVI procedure. In addition, there was a positive correlation between troponin I value and left ventricular mass index before TAVI.

## DISCUSSION

In the present study, we showed that serum apelin levels were significantly lower in symptomatic AS patients compared with the control subjects. Moreover, we found that serum apelin levels were significantly increased one month after TAVI procedure compared to preprocedural levels. Also, we found that there was a significant and negative correlation between serum Pro-BNP and serum apelin levels both before and after the TAVI procedure. To the best of our knowledge, this is the first study to investigate serum apelin levels in symptomatic AS patients before and after TAVI procedure.

Previous studies have shown that serum Pro-BNP level is elevated in patients with AS and serum Pro-BNP level is reduced after TAVI procedure^14,15^. A recent study investigating the level of serum apelin in patients with calcific AS showed an inverse relationship between the severity of calcific AS and serum apelin level^11^.

Narrowing in the left ventricular outflow tract due to AS causes an increase in left ventricular systolic and diastolic pressures, left ventricular muscle mass, and results in left ventricular hypertrophy. If AS is left untreated, the left ventricle enlarges, systolic function deteriorates, and heart failure occurs over time^1^. As a result, serum Pro-BNP levels increase in response to pressure and volume overload in the left ventricular myocardium^1^.

Apelin is an endogenous peptide that is essential for various biological processes and plays an important role in the regulation of cardiovascular functions^8^. In previous studies, serum apelin levels were found to be lower in many cardiovascular diseases such as coronary artery disease, acute myocardial infarction, advanced heart failure, pulmonary HT, and systemic HT when compared with the control group^9,12,16-18^.

In their study, Chong et al. have shown that serum Pro-BNP levels were significantly higher and serum apelin levels were significantly lower in patients with heart failure with reduced left ventricular ejection fraction (HFrLVEF) than in control subjects^12^. Similarly to this study, in another study in which patients undergoing cardiac resynchronization therapy for HFrLVEF were included, a significant decrease in serum Pro-BNP levels and a significant increase in serum apelin levels were observed after cardiac resynchronization therapy^19^. In another study, a significant increase in tissue apelin levels was found after the procedure in HFrLVEF patients who underwent left ventricular assist device^20^.

HT, similarly to AS in terms of pressure overload, can cause left ventricular hypertrophy and diastolic and systolic dysfunction. Previous studies have shown that serum apelin levels are lower in hypertensive patients compared to the control groups^9,21,22^. In a study which included 344 untreated HT patients, serum apelin levels were found to be lower in patients who developed left ventricular hypertrophy than in patients without left ventricular hypertrophy^21^.

In another study, which enrolled HT patients, a significant decrease in systolic and diastolic blood pressure resulted in improvement of diastolic function parameters and an increase in serum apelin levels one month after treatment^22^. In our study, a significant increase in serum apelin levels and a decrease in serum Pro-BNP levels were observed after the TAVI procedure with the removal of left ventricular pressure load.

In our study, left ventricular muscle mass was significantly higher and left ventricular diastolic dysfunction was more prominent in patients with AS compared to the control group. However, there was no significant difference in left ventricular systolic functions between the groups. Previous studies have shown improvement of left ventricular diastolic and systolic functions in patients who underwent TAVI procedure for severe symptomatic AS^23,24^. In our study, left ventricular diastolic parameters improved after TAVI procedure, but there was no significant difference in systolic functions determined by left ventricular ejection fraction (LVEF).

In a recent study, serum troponin I and Pro-BNP levels were significantly higher in patients with AS compared to healthy controls, and serum troponin I values were found to be correlated with left ventricular mass index^25^. Similarly, in our study, serum troponin I values were found to be high in patients with AS and a positive significant correlation was found between left ventricular mass index and troponin I value.

### Limitations

The most important limitation of our study is that it is a single-center study and its sample size is small. Another important limitation was the short follow-up period of the patients, and that it was not possible to determine whether serum apelin values measured before and after TAVI had a role in predicting a clinical event. In our study, although there was an improvement in left ventricular diastolic functions assessed by TTE after TAVI procedure, there was no improvement in systolic functions. However, left ventricular systolic functions were evaluated with LVEF in our study, which is not sufficient. Evaluation of left ventricular systolic functions by speckle tracking echocardiography could be more valuable.

## CONCLUSION

In the present study, serum apelin levels were found to be significantly lower in patients with AS compared to the control subjects. Moreover, serum apelin levels were increased significantly after TAVI procedure in symptomatic AS patients. In addition, a negative and moderate correlation was found between serum apelin and Pro-BNP levels before and after TAVI procedure.

**Table:** 

Authors' roles & responsibilities	
MK	Substantial contributions to the conception or design of the work; or the acquisition, analysis, or interpretation of data for the work; final approval of the version to be published
SS	Substantial contributions to the acquisition, analysis, or interpretation of data for the work
ODU	Substantial contributions to the conception or design of the work; or the acquisition, analysis, or interpretation of data for the work; final approval of the version to be published
AY	Substantial contributions to the conception or design of the work; or the acquisition, analysis, or interpretation of data for the work; final approval of the version to be published
SK	Substantial contributions to the conception or design of the work; or the acquisition, analysis, or interpretation of data for the work; final approval of the version to be published
OS	Substantial contributions to the conception or design of the work; or the acquisition, analysis, or interpretation of data for the work; drafting the work or revising it critically for important intellectual content; final approval of the version to be published
IHK	Final approval of the version to be published
